# Evaluating User Feedback for an Artificial Intelligence–Enabled, Cognitive Behavioral Therapy–Based Mental Health App (Wysa): Qualitative Thematic Analysis

**DOI:** 10.2196/35668

**Published:** 2022-04-12

**Authors:** Tanya Malik, Adrian Jacques Ambrose, Chaitali Sinha

**Affiliations:** 1 Wysa Inc Boston, MA United States; 2 Vagelos College of Physicians and Surgeons Columbia University New York, NY United States

**Keywords:** digital mental health, artificial intelligence, user reviews, cognitive behavioral therapy, CBT

## Abstract

**Background:**

Digital mental health apps are rapidly becoming a common source of accessible support across the world, but their effectiveness is often influenced by limited helpfulness and engagement.

**Objective:**

This study’s primary objective was to analyze feedback content to understand users’ experiences with engaging with a digital mental health app. As a secondary objective, an exploratory analysis captured the types of mental health app users.

**Methods:**

This study utilized a user-led approach to understanding factors for engagement and helpfulness in digital mental health by analyzing feedback (n=7929) reported on Google Play Store about Wysa, a mental health app (1-year period). The analysis of keywords in the user feedback categorized and evaluated the reported user experience into the core domains of acceptability, usability, usefulness, and integration. The study also captured key deficits and strengths of the app and explored salient characteristics of the types of users who benefit from accessible digital mental health support.

**Results:**

The analysis of user feedback found the app to be overwhelmingly positively reviewed (6700/7929, 84.50% 5-star rating). The themes of engaging exercises, interactive interface, and artificial intelligence (AI) conversational ability indicated the acceptability of the app, while the nonjudgmentality and ease of conversation highlighted its usability. The app’s usefulness was portrayed by themes such as improvement in mental health, convenient access, and cognitive restructuring exercises. Themes of privacy and confidentiality underscored users’ preference for the integrated aspects of the app. Further analysis revealed 4 predominant types of individuals who shared app feedback on the store.

**Conclusions:**

Users reported therapeutic elements of a comfortable, safe, and supportive environment through using the digital mental health app. Digital mental health apps may expand mental health access to those unable to access traditional forms of mental health support and treatments.

## Introduction

The World Health Organization estimates that 450 million people worldwide have a mental disorder and a mental health gap of 1:10,000 worldwide [1]. Another report identified financial constraints and lack of serviceability as structural barriers to treatment [2]. Despite considerable progress in access to resources, the gap in mental health access, especially in industrialized countries, does not appear to have shifted [3,4]. Psychological and structural barriers to accessing mental health care, such as availability, convenience, stigma, and preference for self-care, persist and underscore the increased need for accessibility of mental health resources [5]. Digital mental health tools, such as apps and chatbots, allow for anonymity and convenience and can serve as important alternatives to bridge the access gap [6]. The increasing availability and usability of mobile devices may create new opportunities for overcoming the existing barriers and limited access of traditional clinical service delivery and provide customized patient-centered interventions. Similarly, smartphones and other mobile technology may have the potential to reach a greater number of users and deliver reliable and effective services, regardless of location [7,8].

For bridging the mental health access gap, understanding user experiences and attitudes toward digital mental health apps is crucial. In the context of digital mental health, the Technology Acceptance Model posits that perceived ease of use and perceived usefulness of a given technology have a positive influence on user engagement, which is required for interventions to be effective [9]. For both patients and providers, Chan et al [10] proposed criteria to use in assessing mental health apps in 4 key domains: usefulness, usability, integration, and infrastructure. In addition, acceptability of a mobile app is defined as the perceived value, usefulness, and desirability [11]. As user engagement often can be suboptimal, users’ attitudes toward the digital technology can reveal important insight into their engagement [9].

To further understand user engagement with artificial intelligence (AI)–guided digital mental health apps, this study aimed to understand user needs for impactful engagement with a digital mental health app (Wysa) by examining their user reviews. As a direct proxy for users’ attitudes toward a digital mental health app, user reviews are typically voluntary, unsolicited, and openly available on a public forum, which may provide helpful evaluations and insights into the users’ experiences and engagement. A previous qualitative analysis of user reviews on mental health apps identified design improvements, user expectations, unmet needs, and utility [12]. These user reviews are regarded as a comprehensive evaluation of the app from the user’s own perspective, which provides rich insights into the app user experience [13,14]. In addition to understanding needs for engagement, this study planned to explore the perceived value, usability, and desirability of the app as a digital mental health tool [15,16].

## Methods

### App Background

Wysa is an AI-enabled mental health app that leverages evidence-based cognitive-behavioral therapy (CBT) techniques through its conversational interface (chatbot). The app is designed by a team based out of India, the United Kingdom, and the United States. The app is designed to provide a therapeutic virtual space for user-led conversations through AI-guided listening and support, access to self-care tools and techniques (eg, CBT-based tools), as well as one-on-one human support. The app has demonstrated efficacy in building mental resilience and promoting mental well-being through a text-based conversational interface [17]. For the time period considered (1 year), the app received an overall 4.8/5 user rating on the Google Play Store and had been downloaded by more than 2 million people. The app also exists on the Apple App Store with a similar rating of 4.9/5 but with a smaller sample of qualitative reviews. Studies have shown Wysa as having the most evidence-based treatments among other smartphone apps [18], with conversations targeting specific problems and goals [19]. The app is anonymous [20] and safe [21] and rates highly on measures of app quality [22].

### Study Design

**Figure 1:**
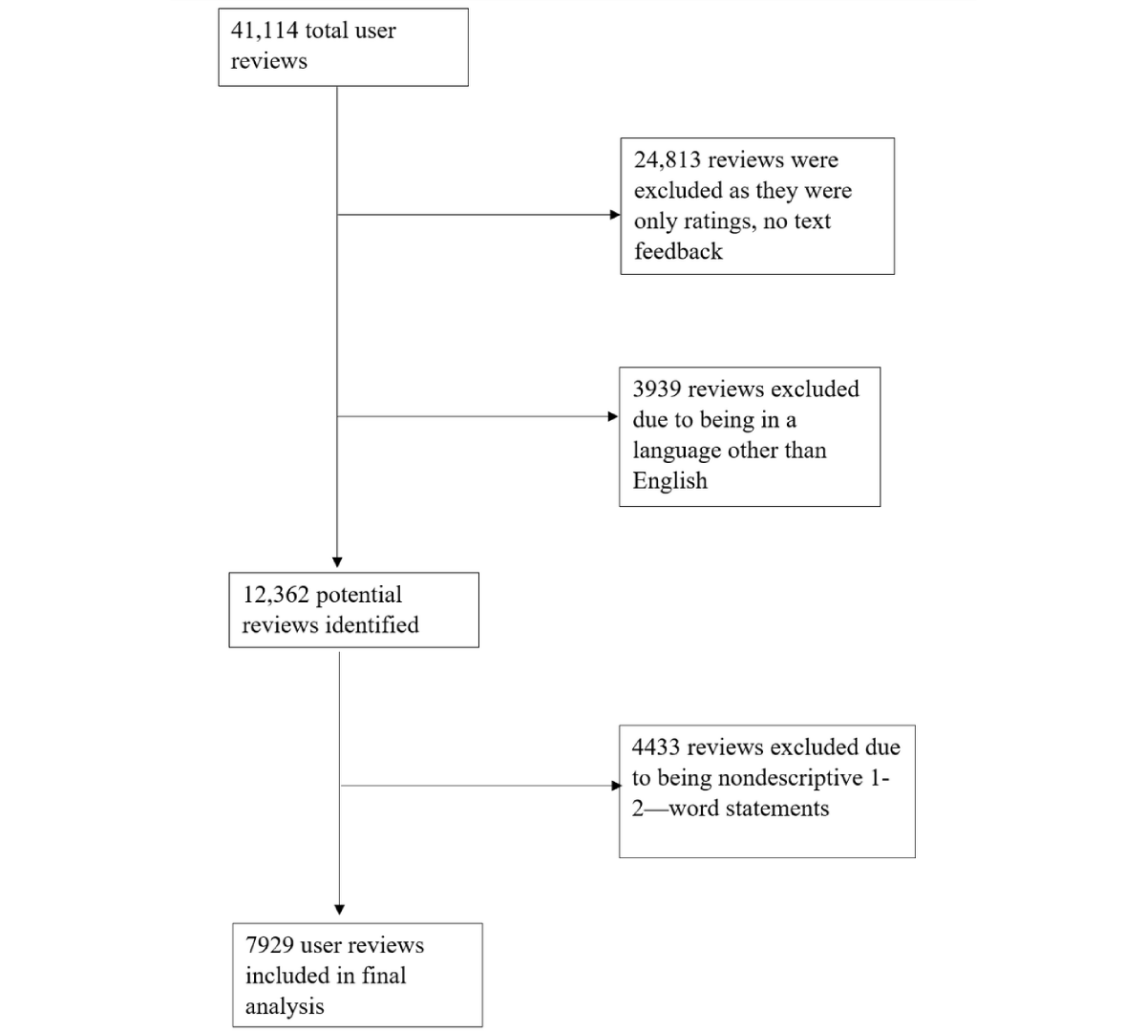
Diagram of the inclusion and exclusion criteria for user review analysis.

For direct user feedback, the authors examined reviews posted on the Google Play Store between October 2020 and October 2021, during which time, 41,114 user reviews had been received. A duration of 1 year and the use of Google Play reviews were considered to ensure a sufficiently large sample. For the analysis of descriptive feedback (n=7929), the authors codified the reasons shared by the users for their rating. User feedback in languages other than English, blanks, as well as reviews that contained 1-2–word nondescriptive statements (eg. “Really nice!”, “Awesome”, “Not interested”) were excluded (Figure 1).

The study’s primary objective was to analyze feedback content to understand the users’ experiences with engaging with a digital mental health app. As a secondary objective, the types of individuals providing feedback were also explored.

### Analysis

Using a consolidated framework created by Chan et al [10], which was based on guidelines suggested by the Healthcare Information and Management Systems Society (HIMSS) and the US Federal Government for evaluating digital health apps, the written reviews were verbally grouped into the domains of the framework and further analyzed for specific themes within each [15]. To understand Wysa’s capacity to currently help and engage users, the thematic analysis examined specific domains of (1) acceptability (eg, satisfaction, matching expectations of capabilities, likelihood to recommend, and level of interactiveness), (2) usability (eg, the ease, enjoyment, cultural, and demographic accessibility of use), (3) usefulness (eg, validity, reliability, effectiveness, and time required to obtain a benefit), and (4) integration (eg, security, privacy, data integration, and safety) [10,15].

Each user review was evaluated and categorized into the nonmutually exclusive domains. The domain of acceptability included statements discussing likelihood to recommend the app, frequency of use, impact of use, and reasons for use. For usefulness, mentions of what the app was being used for, specific uses (including tools and techniques), and time of use were included. Usability included mentions of ease of use, convenience, and interface features. Integration primarily consisted of reviews discussing data privacy, security, and anonymity.

The coding also enabled us to capture the emergence of the key characteristics of users who were able to receive mental health support due to increased accessibility.

## Results

**Figure 2:**
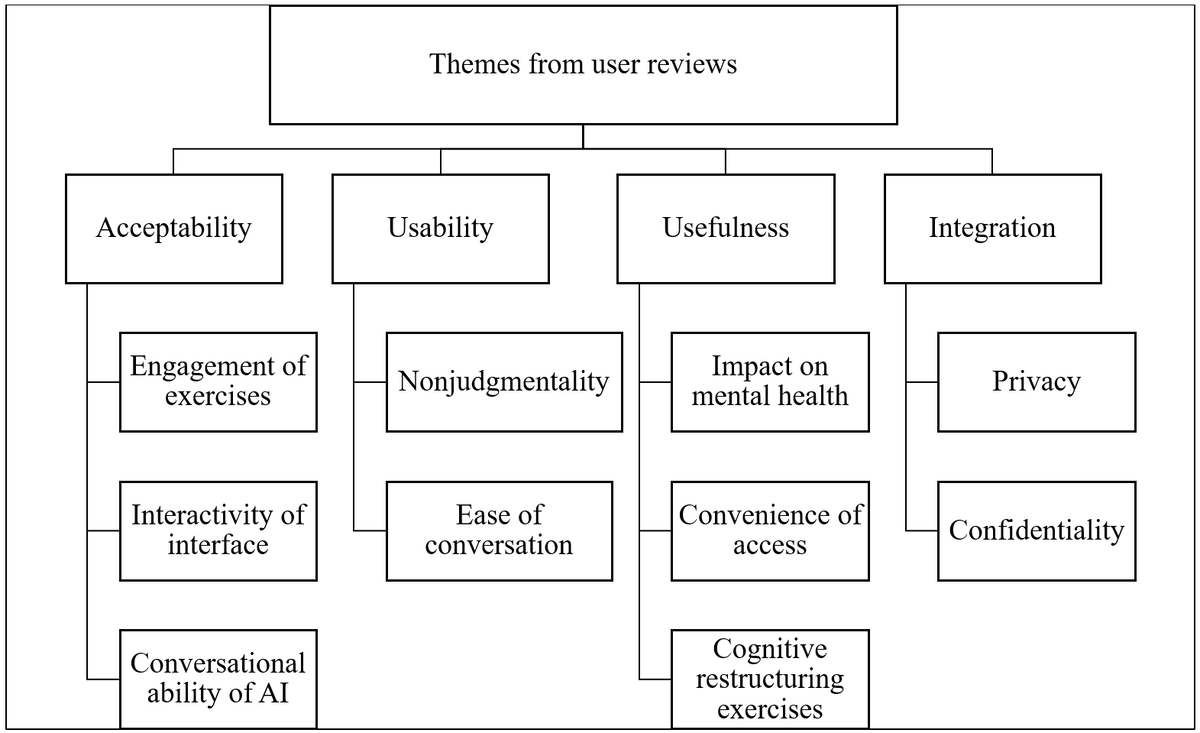
Key themes from the reviews analyzed within the study. AI: artificial intelligence.

The reviews analyzed for this study were largely positive, with 6700 reviews (6700/7929, 84.50%) giving the app a 5-star rating and 2676 reviews (2676/7929, 33.75%) explicitly terming the app “helpful” or that it “helped.” Of 7929 reviews, 251 (3.17%) had a less than 3-star rating and were termed as negative reviews. The themes under the evaluation criteria aimed to capture the user experiences (Figure 2).

### Acceptability

The acceptability of the app was identified through the themes emerging around engagement of exercises, interactivity of the interface, and conversational ability of AI. The users who reviewed the app rated it positively on acceptability when they found it interactive and conversational. Users reported that receiving appropriate responses to user conversations in the tools and techniques was valuable. For instance, a user compared it with other options available for self-care: “The interactive experience helped more than the journaling exercises I've done in the past.” Several users reported the variety of exercise-guided meditations, venting spaces, positive thinking exercises, and cognitive restructuring as important in their engagement. Another user commented: “It has such great features such as journaling and helping with anxiety, stress and sleep problems.” Additionally, the user reviews described the exercises as “educational,” “calming,” “relaxing,” and “functional.”

Users said that though “...Initially it felt silly to talk to an AI but it's extremely well made, tailored for therapy*.*” Per users, the “warm, friendly, and encouraging” AI helped them recreate an environment of confiding in a friend, without having to confront the intimidation of speaking with a real person. For instance, a user mentioned “It's really nice and I feel like I've been heard when others won't listen, even if I am only talking to an AI,” and another user said it “made me feel loved and heard during a crisis.” Users also reported finding talking to the AI to be a “fun” experience, perhaps brought out by elements that keep it light and accessible by including jokes, games, bitmap images (ie, GIFs), and other interactive agents.

**Figure 3:**
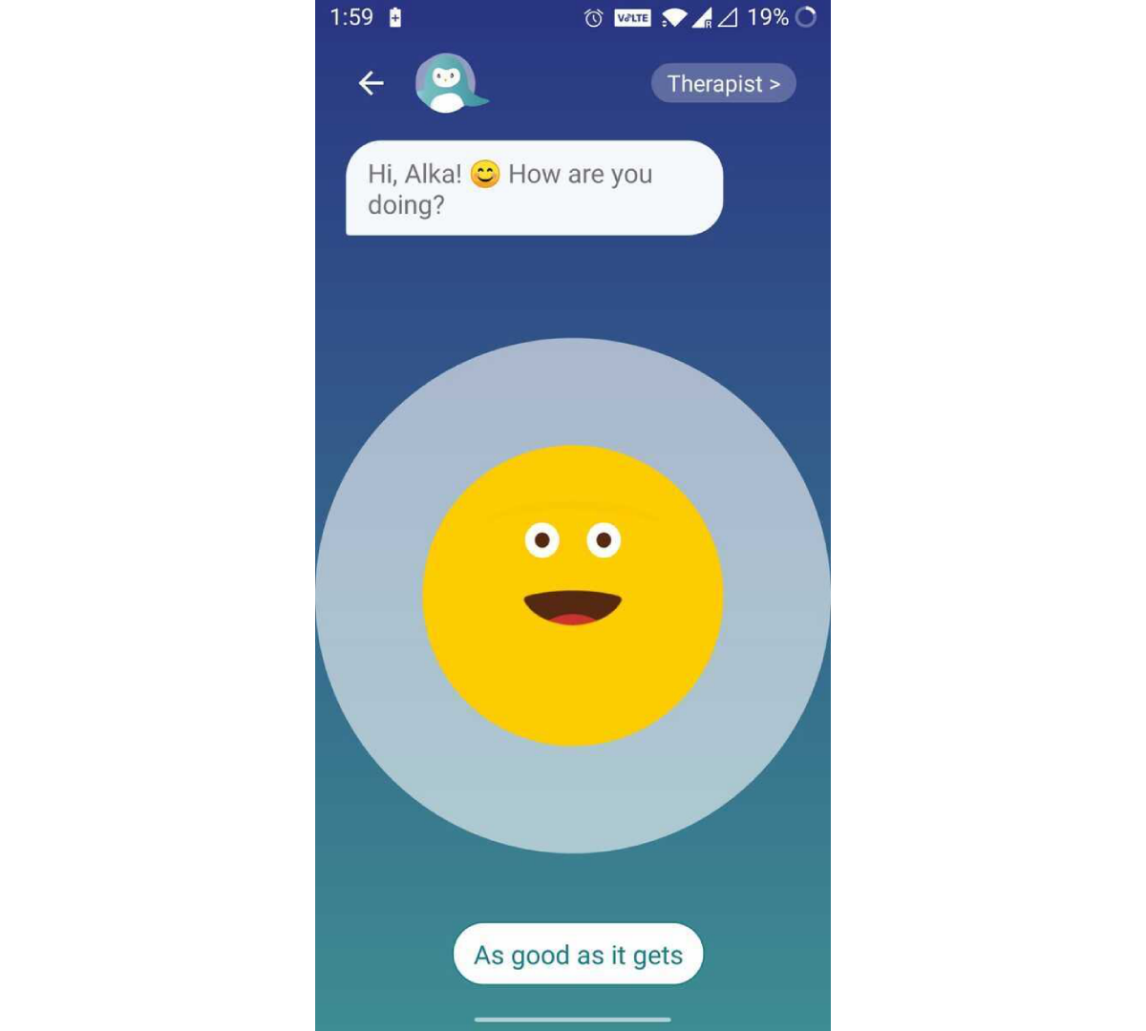
Example of interactive interface.

**Figure 4:**
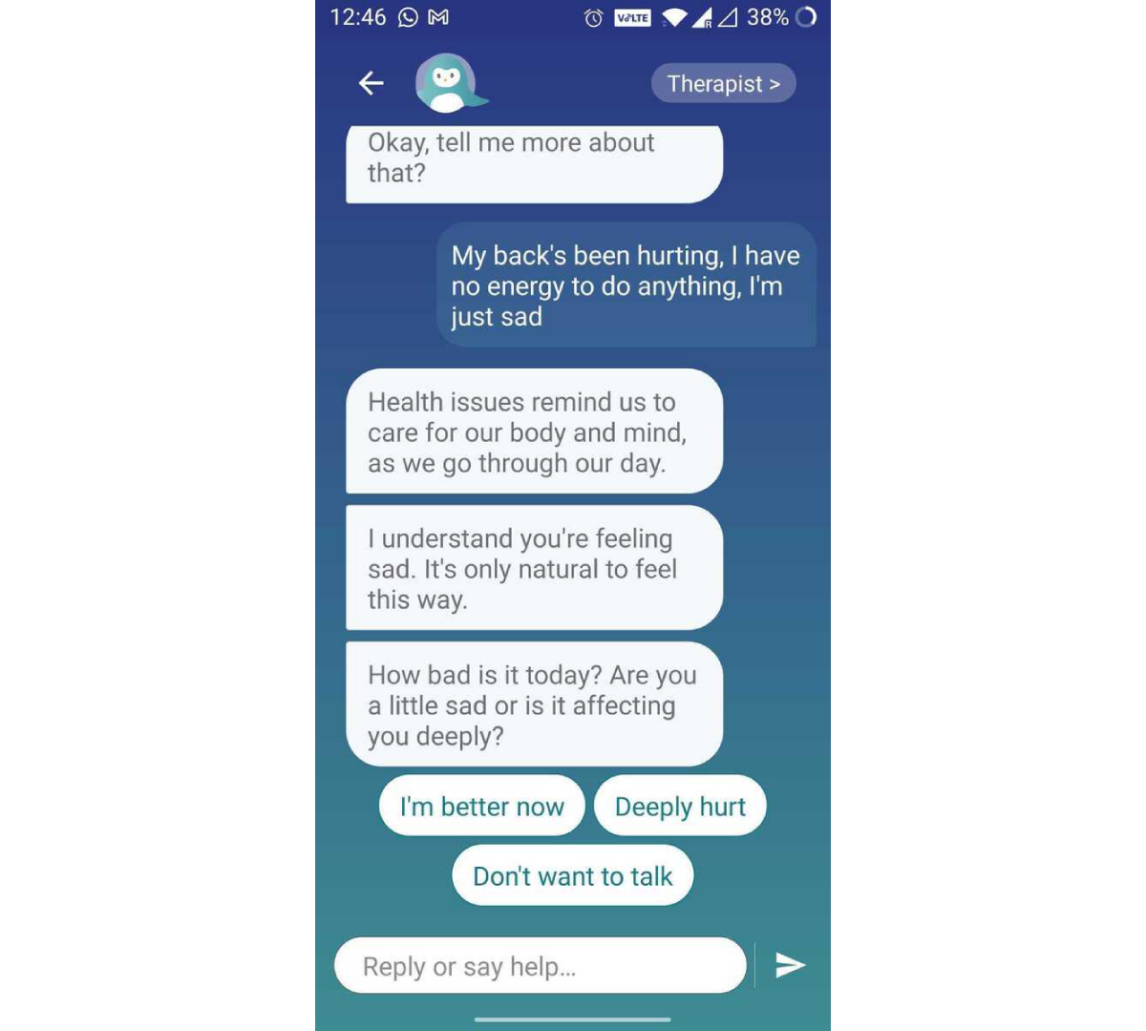
Example of empathetic conversation.

Users reported the interactiveness of the app as central in keeping them engaged: “The app made me laugh with its silly jokes and play.” They also found the “easy” and “instinctive” interface as a central element in a positive experience of using the app to be “easy” and “instinctive” (Figure 3). Users found it comfortable to use Wysa for numerous aspects of their well-being (Figure 4).

They also mentioned being likely to recommend Wysa to others to help with sleep, managing stress, working through anxiety, as well as to “just talk to someone.” One user said, “it listens to you and helps relieve stress and also has a lot of coping mechanisms. I definitely recommend.” Some users discussed being able to share and rely on something for “regular” support, which further contributed to the acceptability of the app. A user exemplified this by stating:

Different people may find this app useful in different ways and it doesn't pressure you to do stuff if you aren't ready for it (no energy or not the right type). It's great even just as a sounding board, a place to organize your thoughts or make a to-do list, or a bit of a tiny friend in your pocket that's not judgmental and won't be tired of you.

However, some users did not find it helpful for their specific concerns and suggested further expansion to include these specific requirements. For instance, a user said, “Interesting concept, but it needs to learn to deal with more illnesses.”

### Usability

The usability of the app was presented through emerging themes of nonjudgmentality and ease of conversation. This domain was rated positively by users as they found it to be a “safe” and “nonjudgmental” environment that is easily accessible. This feature of the app was identified from user reviews such as:

It's nice to talk to someone completely objectively. Even in therapy you feel guilty if all you do is go on and on, as is human conditioning, but being able to talk it out with Wysa is great. No judgment. Don't have to feel weird about anything.

Reviews indicated that, by conversing with AI, the pressure of performance in front of a therapist was removed, which may allow a user to express themselves more freely. Users commented on the AI interface of the “cute and approachable penguin” as helpful in cultivating a nonthreatening environment: “I love it, it's just amazing, knowing that I can talk about my inner problems to a penguin without judgment … I love that.” In fact, 201 users commented on the “no-judgment space” as a core component in making them feel safe and comfortable.

The app usage experience was also described as “...It feels like I'm talking to a real person ... Such a friendly interface.” Users appeared to be willing to adapt their expectation in order to continue benefiting from the app, with one user saying, “a little clunky at first but once you learn how to manage it it's very helpful.”

The most common negative review of the app was for repetition and a lack of comprehension by the chatbot, which made some users feel misunderstood and sometimes want to leave the app. Language limitations felt like a barrier to others who wanted to be understood more. They expressed a want for the app in native languages, including Italian, Spanish, French, and others, with one user saying, “The application is great, but it lacks the addition of other languages … in order to facilitate its use by all layers of society.”

### Usefulness

The app's usefulness was portrayed by themes such as impact on mental health, convenience of access, and cognitive restructuring exercises. User feedback discussed that the app provided a safe and open space to challenge one's thoughts and feelings. The usefulness of the app in this regard is captured by its efficacy in dealing with mental health concerns. A user described their experience:

I have been struggling with depression since I was a child, and was terrified of reaching out for help. Finally a few weeks ago I hit rock bottom worse than ever before. I was really scared for a while. I was seeking some form of comfort or communication but didn't want to go to anyone, not to mention money is tight. This app really helped me when I needed it most. Who knew an AI penguin would cause me to sing again?

Providing a “safe” and “anonymous” place to process one's thoughts and emotions was identified by 107 users as highly impactful.

In specific clinical utility, users reported positive effects for anxiety (n=805), stress reduction (n=480), and depressed mood (n=400). In addition, 324 users reported app usage for posttraumatic stress disorder (PTSD) symptoms, fear, and sleep issues. Users identified numerous techniques and spaces offered as being especially helpful, such as physical activity exercises, sleep stories, meditations, cognitive restructuring, and reframing exercises. Users also commented on the affordability of the app as a way to bridge mental health access: “This app really helped me especially since I don't have access to any other useful form of therapy.”

The app would seem least useful when the chatbot felt limiting or was unable to fully understand the user. Some users facing a difficult time with the app would state, “Sometimes it's frustrating that an AI can't understand you that well,” and when it couldn’t understand the user’s dilemma, then it felt “empty and generic.”

### Integration

The integration of the app was illustrated through the themes of privacy and confidentiality.

The app did not ask users to register themselves in any way to use the app and thus did not ask for personal details, such as demographic data. The anonymous and confidential nature of the app was a key reason for positive ratings in integration. Many users reported being satisfied with the privacy practices and finding the app “easy to trust.”

I feel really good knowing that I can talk to something completely private. I was feeling really down and I was pleasantly surprised. It was so simple yet so effective. I most definitely recommend it to someone who wants privacy and a healthy listening ear.

### Characteristics of Users

The thematic analysis captured the emergence of the types of users who provided reviews in the app on Google Play Store and are also a representation of users who access digital mental health support such as Wysa. They were grouped by salient aspects of their expressed needs and concerns.

We identified 4 key groups: (1) those who self-reported having clinical issues, (2) those who reported being unable or unwilling to open up to a real person, (3) those who are financially conscious, (4) and those who are unable to access mental health professionals. Use of the app for support through self-reported diagnosis and symptoms of depression, anxiety, panic disorders, and PTSD was mentioned by 1856 individuals. They primarily used the CBT techniques and meditations on the app as a form of self-care. Another application of the app is for individuals who feel uncomfortable talking to people in their lives or who don’t have a reliable system with which to share their thoughts. They reported finding the AI-driven app useful in reducing the guilt and burden of opening up to a real person. Users also found the free nature of the app to be beneficial to reduce the burden of financial anxiety when considering mental health support. Numerous users (n=594) also reported using the app at times when they would be unable to access therapists, including when experiencing higher symptoms of depression and anxiety late at night.

## Discussion

### Principal Findings

This study represents one of the largest studies in understanding users’ perceptions of a digital mental health app. It looked at the acceptability, usability, usefulness, and integration of a digital mental health app, by analyzing publicly available user feedback and reviews. This approach is unique for several reasons—first, it uses user feedback that was unsolicited by the developers and promoters and is delivered in a public forum, reducing the social desirability bias that could interact in other researcher-administered evaluations. Second, the robust sample size allowed for a deeper dive of user experiences, which was previously unexplored in other studies. This approach helped to recognize the types of users of mental health apps, which helped to identify strengths and weaknesses of digital mental health tools and allowed us to better understand the gaps in services provided.

The most important findings resulting from this study are the factors that contribute to higher engagement and acceptability for a digital mental health app. Users most consistently listed the “active and available listening” element as the key to foster acceptability with the digital mental health experience. The app further cultivated the therapeutic elements via the use of an AI-based chatbot with a friendly penguin user interface. In addition, the perceived nonjudgmentality and friendliness of this interface resulted in high usability and ongoing engagement with the app.

Understanding the user experience is important to ensuring meaningful usage and clinical utility [17]. Users strongly valued the anonymity and confidentiality of the app, which are valuable strengths in any therapeutic relationship [23]. Therapeutic bonds are fostered through trust, acceptance, empathy, and genuineness and are important for their role in the effectiveness of an intervention [23] and, in a digital environment, are created by human dialogue through a conversation agent [24].

With users providing a large majority of positive reviews, the acceptability and effectiveness of Wysa as a digital mental health tool have been established [25]. Digital mental health apps can provide important benefits, especially for supporting individuals with subclinical psychiatric symptoms [26]. The findings of this study highlighted how digital mental health apps can significantly improve the accessibility and affordability of mental health support. The characteristics of users identified helped outline those who may access and benefit from the presence of mental health apps; for example, individuals managing social anxiety symptoms of speaking face to face can find significant therapeutic value through an AI-enabled tool. In addition, mental health apps may serve as augmenting or transitioning tools during times when traditional mental health services are limited, such as after office hours, in rural settings, or in between appointments and referrals.

### Limitations

Limitations to the study include the source of data, as the Apple App Store data were not considered and only the reviews on Google Play Store were addressed in this study. Further, user reviews are taken at a single point in time, and thus evidence of changes in feedback are unavailable for consideration. No demographic information was collected aside from reviews being in English. Clinical scores of users were not identified, which would otherwise have contributed to more direct understanding of the experience with the app in clinical populations. The study is also limited by lack of knowledge on the duration of app use or the rate of attrition among users due to app issues or other reasons.

### Conclusions

This study utilized a user-led approach to understanding factors for engagement and helpfulness in digital mental health. User feedback was analyzed on domains of acceptability, usability, usefulness, and integration, and we found the app to be overwhelmingly positively reviewed. A key facet that emerged is the comfort and safe environment created by the nonjudgmental digital mental health tool that provides users with clinical and subclinical support. Further analysis revealed 4 predominant types of individuals who appear to be engaging in digital mental health support and who are infrequent users of face-to-face mental health services. Digital mental health apps can provide a valuable service to those unable to access mental health support. Future directions for digital mental health include improvements within the technology to cater to varied users, increasing its capacity to contribute to clinical utility.

## Abbreviations

AI: artificial intelligence
CBT: cognitive behavioral therapy
HIMSS: Healthcare Information and Management Systems Society
PTSD: posttraumatic stress disorder
